# A 2.8-Å resolution structure of the skatole-producing glycyl radical enzyme Indoleacetate Decarboxylase enabled by new techniques in cryo-EM grid preparation.

**DOI:** 10.1063/4.0000922

**Published:** 2025-10-27

**Authors:** Christa N Imrich, Lindsey RF Backman, Mary C Andorfer, Catherine L Drennan

**Affiliations:** Massachusetts Institute of Technology, Cambridge, MA, USA

## Abstract

Nature has devised an array of enzymatic cofactors that enable challenging chemical transformations to occur on a timescale compatible with biological life. Protein-based amino acid radicals are one example of a simple yet powerful radical cofactor capable of catalyzing diverse chemistry. The glycyl radical enzyme (GRE) superfamily is a prominent example of this type of cofactor and the catalytic power it possesses. GREs use a radical housed on the a-carbon of a glycine residue in the active site to perform catalysis in anaerobic environments. The radical- storing glycine residue is housed in the glycyl radical domain (GRD), which is found at the C-terminus of the polypeptide and must undergo a large conformational change to flip out of the active site for radical installation. This radical must be post-translationally installed by a radical *S*- adenosyl-L-methionine (rSAM) activating enzyme that is specific to each GRE. Therefore, the GRD is intrinsically highly conformationally dynamic. Some GREs employ additional subunits or secondary structures to both cover the active site and contact the GRD, which keeps the enzyme in a closed state. However, additional dynamic subunits and regions can further complicate structural determination. Initial attempts to structurally characterize the GRE Indoleacetate Decarboxylase (IAD) by both X-ray crystallography and cryo-electron microscopy (cryo-EM) resulted in structures with the active site in an open, disordered conformation. The rapid expansion of the cryo-EM field in recent years has provided new tools for sample preparation, data collection, and analysis. The SPT Labtech chameleon is a non-blotting, alternative grid preparation technique that has been demonstrated to ameliorate air-water interface denaturation and enable the capture of low-occupancy oligomeric states. Here, we utilize the chameleon for cryo-EM grid preparation which has enabled the capture of a high-resolution reconstruction of a tetrameric IAD from the gut microbe *Olsenella uli* to 2.8-Å resolution in a closed, ordered conformation with the substrate indole-3-acetate bound in the active site.

